# Endemic Human Coronavirus Antibody Levels Are Unchanged after Convalescent or Control Plasma Transfusion for Early Outpatient COVID-19 Treatment

**DOI:** 10.1128/mbio.03287-22

**Published:** 2023-01-10

**Authors:** Andrew H. Karaba, Trevor S. Johnston, Evan Beck, Oliver Laeyendecker, Andrea L. Cox, Sabra L. Klein, David J. Sullivan, COVID-19 Serologic Studies Consortium

**Affiliations:** a Department of Medicine, Division of Infectious Diseases, Johns Hopkins University School of Medicine, Baltimore, Maryland, USA; b Division of Intramural Research, National Institute of Allergy and Infectious Diseases, NIH, Baltimore, Maryland, USA; c The Department of Molecular Microbiology and Immunology, Johns Hopkins Bloomberg School of Public Health, Baltimore, Maryland, USA; Anne Arundel Medical Center; Ascada Research; Ascada Research; Baylor College of Medicine; MedStar Georgetown University Hospital; Johns Hopkins Center for American Indian Health; Johns Hopkins Center for American Indian Health; Johns Hopkins Bloomberg School of Public Health; Johns Hopkins Bloomberg School of Public Health; Johns Hopkins Bloomberg School of Public Health; Johns Hopkins Bloomberg School of Public Health; Johns Hopkins Bloomberg School of Public Health; Johns Hopkins Bloomberg School of Public Health; Johns Hopkins Bloomberg School of Public Health; Johns Hopkins Bloomberg School of Public Health; Johns Hopkins Bloomberg School of Public Health; Johns Hopkins University; Johns Hopkins University; Johns Hopkins University; Johns Hopkins University; Johns Hopkins University; Johns Hopkins University; Johns Hopkins University; Johns Hopkins University; Johns Hopkins University; Johns Hopkins University; Johns Hopkins University; Johns Hopkins University; Johns Hopkins University; Johns Hopkins University; Lifespan/Brown University Rhode Island Hospital; Mayo Clinic, Phoenix; MedStar Washington Hospital Center; NorthShore University HealthSystem; NorthShore University HealthSystem; The Bliss Group; The Next Practice Group; University of California Los Angeles; University of Alabama at Birmingham; University of California, Irvine Health; University of California, San Diego; University of California, San Diego; University of Cincinnati Medical Center; University of Massachusetts Worcester; University of Miami; University of New Mexico; University of Rochester; University of Texas Health Science Center at Houston; University of Utah Health; University of Utah Health; Vassar Brothers Medical Center; Vassar Brothers Medical Center; Wayne State University; Western Connecticut Health Network, Danbury Hospital; Western Connecticut Health Network, Danbury Hospital; Western Connecticut Health Network, Danbury Hospital; Western Connecticut Health Network, Danbury Hospital; Western Connecticut Health Network, Norwalk Hospital; Western Connecticut Health Network, Norwalk Hospital; Albert Einstein College of Medicine

**Keywords:** COVID-19, SARS-CoV-2, endemic human coronaviruses, plasma transfusion

## Abstract

The impact of preexisting antibodies to the four endemic human coronaviruses (ehCoV) (229E, OC43, NL63, and HKU1) on severe (hospitalization) coronavirus disease 2019 (COVID-19) outcomes has been described in small cohorts. Many studies have measured ehCoV 229E, OC43, NL63, and HKU1 antibody levels weeks after recovery rather than in the first weeks of illness, which is more relevant to early hospitalizations. Antibody levels to the spike protein of the four coronaviruses (229E, OC43, NL63, and HKU1), as well as severe acute respiratory syndrome coronavirus 2 (SARS-CoV-2), were measured both before and immediately after convalescent or control plasma transfusion in 51 participants who were hospitalized and 250 who were not hospitalized, as well as in 71 convalescent and 50 control plasma donors as a subset from a completed randomized controlled trial. In COVID-19 convalescent plasma donors, the ehCoV spike antibodies were 1.2 to 2 times greater than the control donor unit levels, while donor COVID-19 convalescent plasma (CCP) SARS-CoV-2 spike antibodies were more than 600 times the control plasma units. Plasma transfusion, whether COVID-19 convalescent or control, did not alter the post-transfusion antibody levels for the endemic human coronaviruses (229E, OC43, NL63, and HKU1) in those hospitalized and not hospitalized, despite the 1.2- to 2-fold elevation in donor COVID-19 convalescent plasma. There was no influence of prior antibody levels to 229E, OC43, NL63, and HKU1 or post-transfusion antibody levels on subsequent hospitalization. These data, from a well-controlled prospective randomized clinical trial, add evidence that antibodies to ehCoV do not significantly impact COVID-19 outcomes, despite the apparent back-boosting of some ehCoV after SARS-CoV-2 infection.

## OBSERVATION

The influence of preexisting antibodies to the four endemic human coronaviruses (ehCoV) (229E, OC43, NL63, and HKU1) on severe (hospitalization) coronavirus disease 2019 (COVID-19) outcomes has not been fully characterized (1). COVID-19 convalescent plasma (CCP) obtained weeks after illness contains higher IgG levels to the four ehCoV than pre-COVID-19 control plasma (2, 3). Recovered convalescent individuals with severe COVID-19 also have higher antibody levels to severe acute respiratory syndrome coronavirus 2 (SARS-CoV-2), as well as a boosted response to the four ehCoV (4, 5). SARS-CoV-2 vaccination also boosts ehCoV antibodies (6, 7). Most studies measure ehCoV antibody levels weeks after symptom onset instead of in the first week of illness (3, 5). Some studies implicate the cross-reactive protection of ehCoV antibodies (8). Pre-COVID-19 plasma has been shown to neutralize SARS-CoV-2 (9, 10). Recent endemic human coronavirus infection was also associated with less severe COVID-19 (11).

The prospective association of severe COVID-19 with elevated ehCoV antibody levels at illness onset has not been investigated (12). A clinically relevant question was raised recently as to whether antibody levels to ehCoV in control plasma as opposed to saline could deleteriously influence hospital outcomes in controls during a clinical trial (13). The literature has mainly looked retrospectively for correlations of high antibody levels weeks after hospitalization, even though COVID-19 hospitalization itself raises antibody levels to both ehCoV and SARS-CoV-2 (3, 4).

This substudy of a larger trial (14) focuses on the impact of preexisting ehCoV antibody immunity on the outcome of severe SARS-CoV-2 disease by measuring ehCoV antibody levels in the first week of COVID-19, rather than taking a retrospective look back weeks after recovery. Additionally, we examined ehCoV and SARS-CoV-2 antibody levels from unused CCP and nonconvalescent control donor units, as well as before and after randomized transfusion (within the first 9 days of COVID-19), in a representative sampling of participants from a subset of an interventional randomized clinical trial, which demonstrated efficacy at outpatient hospital reduction (14).

Antibodies that bind the spike proteins from 229E, NL63, OC43, HKU1, and SARS-CoV-2 were measured using the Meso Scale Diagnostics (MSD; Rockville, MD) V-PLEX COVID-19 coronavirus panel 3 IgG kit at a dilution of 1:5,000. Plates were read on a Meso QuickPlex SQ 120 instrument, and the arbitrary units (AU) were calculated using the MSD Discovery Workbench software according to the manufacturer’s protocol. For the samples in the spike competition experiment, purified recombinant full-length WA-1 SARS-CoV-2 spike protein (150 μg/mL) (4) was incubated 1:1 with plasma samples for 45 min prior to antibody measurement via the MSD assay as described above. Approvals were obtained from the institutional review boards at Johns Hopkins University School of Medicine under a single IRB (IRB00247590) for all participating sites and the Department of Defense (DoD) Human Research Protection Office. All the trial participants provided written informed consent for the scope of this research.

The nonhospitalized subset (*n* = 250) randomly chosen for endemic human CoV antibody analysis was similar to the full study with regard to key demographic features. This subgroup had a similar percentage of female participants, with similar comorbidities (hypertension, lung disease, diabetes) and a small 4-year mean age difference (see Table S1 in the supplemental material). SARS-CoV-2 vaccination at enrollment was excluded for this substudy.

10.1128/mbio.03287-22.9 TABLE S1 Characteristics of substudy participants compared to those in the parent study (14). Download Table S1, DOCX file, 0.02 MB. Copyright © 2023 Karaba et al. 2023 Karaba et al. https://creativecommons.org/licenses/by/4.0/ This content is distributed under the terms of the Creative Commons Attribution 4.0 International license.

**FIG 1:**
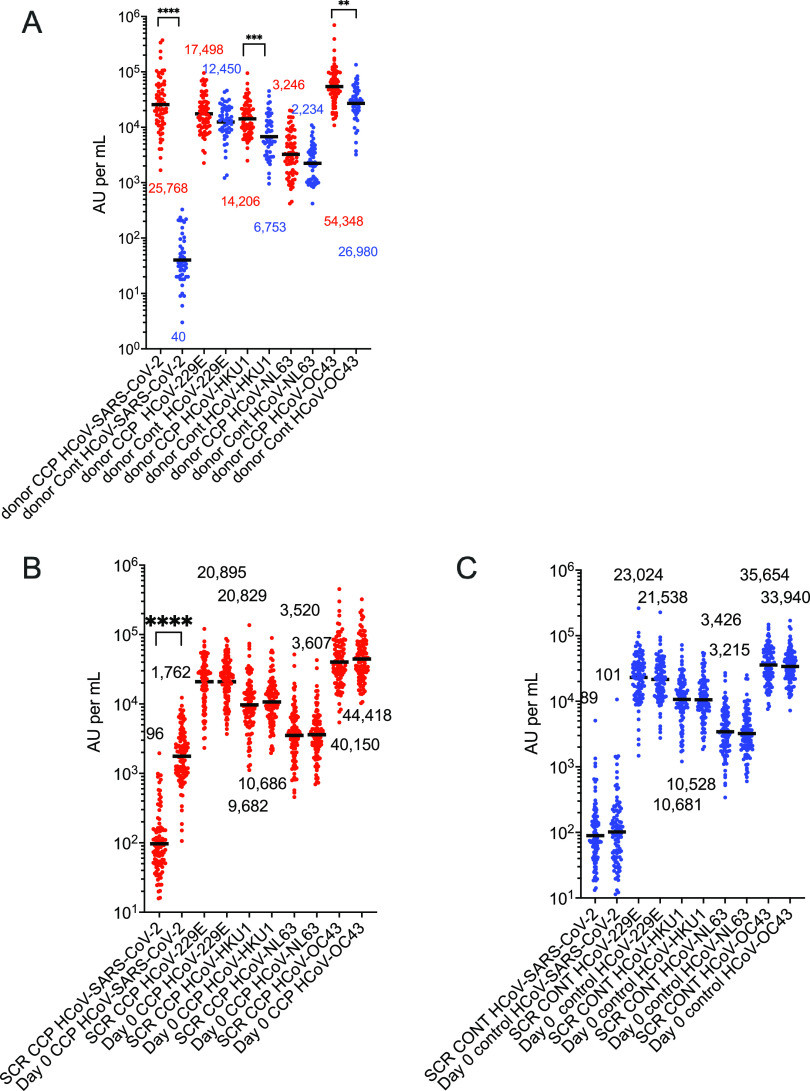
ehCoV antibody levels in donors and nonhospitalized recipients in the early treatment outpatient CCP study. (A) Coronavirus antibody levels (arbitrary units [AU] per milliliter) shown in donor CCP (red circles; *n* = 71) and control plasma (blue circles; *n* = 50) units. CCP ehCoV antibodies were 1.2 to 2 times higher than in control plasma units. SARS-CoV-2 antibody levels were more than 600 times higher in CCP than in control units. (B) Coronavirus antibody levels before and 30 min after transfusion of CCP among participants who were negative for SARS-CoV-2 RBD at enrollment (red circles; *n* = 100). (C) Coronavirus antibody levels before and 30 min after transfusion of control plasma among participants who were negative for SARS-CoV-2 RBD at enrollment (blue circles; *n* = 100). ehCoV antibody levels were similar before and 30 min after transfusion for those transfused with CCP or control plasma. Horizontal black bars represent geometric means. Statistically significant 2-way analysis of variance (ANOVA) (Tukey *post hoc*) of log-transformed values indicated by brackets. *P* values of <0.05 were considered significant. **, *P* = 0.001, ***, *P* = 0.0004, ****, *P* < 0.0001.

**FIG 2:**
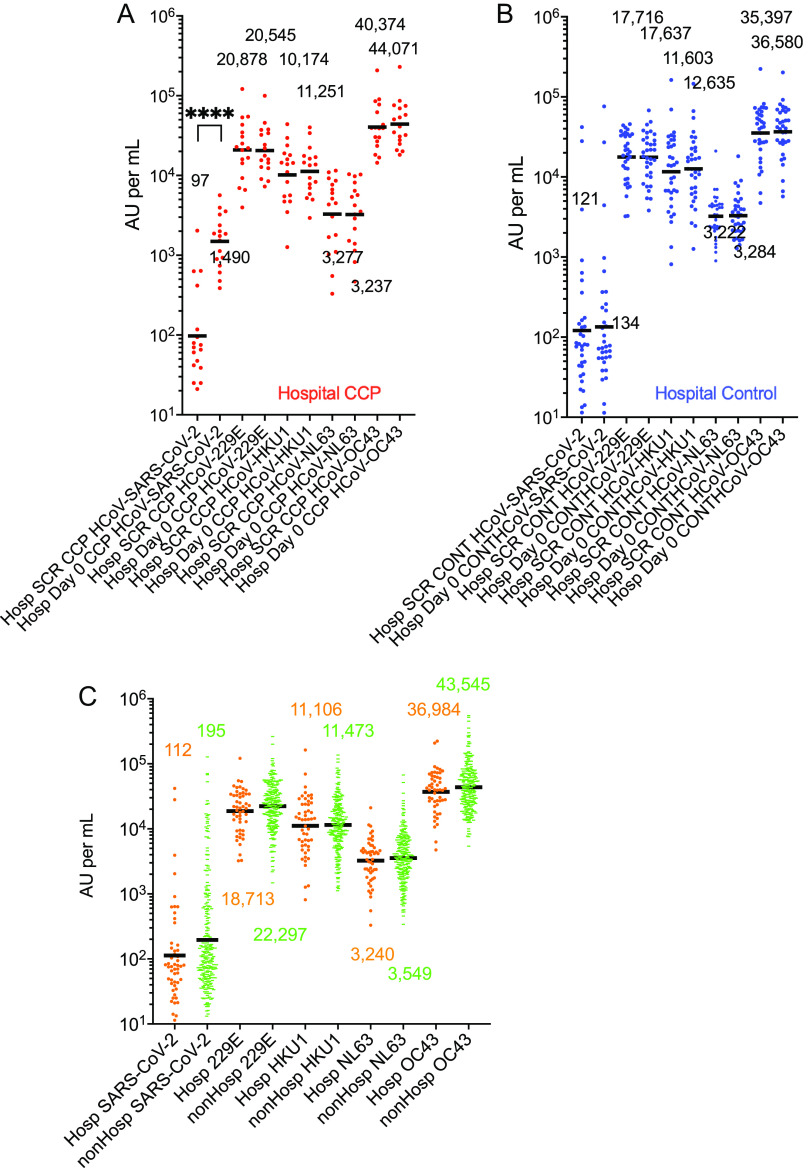
Endogenous human coronavirus (ehCoV) antibody levels in hospitalized participants in the early treatment outpatient CCP study. (A) Antibody levels (AU per milliliter) before and 30 min after transfusion of CCP in participants who were eventually hospitalized (red circles; *n* = 17). (B) Antibody levels in participants who received control plasma and were eventually hospitalized (blue circles; *n* = 34). Except for a SARS-CoV-2 spike in the CCP recipients, all before and after transfusion antibody levels were similar. (C) Antibody levels at time of enrollment in participants who were hospitalized (*n* = 51; orange circles) were compared with antibody levels at enrollment for nonhospitalized participants (*n* = 250; green). No significant differences were observed. Geometric means are indicated with horizontal black bars. Brackets between groups indicate statistically significant differences as determined by 2-way ANOVA (Tukey *post hoc*) of log-transformed values. *P* values of <0.05 were considered significant. ****, *P* < 0.0001.

In CCP donors, the ehCoV spike antibodies were 1.2 to 2 times greater than the control donor unit levels, while the donor CCP SARS-CoV-2 spike antibodies were more than 600 times the levels of the control units (Fig. 1A). In the SARS-CoV-2 CCP participants who were seronegative for the SARS-CoV-2 receptor-binding domain (RBD) by Euroimmun assay, the SARS-CoV-2 spike antibodies increased 17-fold from pretransfusion screen to post-transfusion, while the HKU1 and OC43 antibodies showed a small insignificant increase. In separate seronegative control recipients, the ehCoV antibodies pretransfusion to post-transfusion were also unchanged (Fig. 1B, C). We also measured antibody levels pre- and post-transfusion in 50 participants who were seropositive at enrollment (Fig. S1A, B). Baseline pretransfusion SARS-CoV-2, HKU1, and OC43 antibody levels in seropositive CCP and controls were increased compared to seronegative participants, with no change post-transfusion for all viruses. In the hospitalized CCP and control plasma participants, the pretransfusion and post-transfusion geometric mean antibody levels were similar, except for SARS-CoV-2 in CCP recipients (Fig. 2A, B). A comparison of pretransfusion antibody levels in all 51 hospitalized participants to the levels in those not hospitalized indicated no differences in ehCoV levels (Fig. 2C).

10.1128/mbio.03287-22.1 FIG S1 (A) Coronavirus antibody levels (AU/mL) before and 30 min after transfusion of CCP in participants who were seropositive for SARS-CoV-2 RBD prior to transfusion (*n* = 25; red). (B) Coronavirus antibody levels (AU/mL) before and 30 min after transfusion of control plasma in participants who were seropositive for SARS-CoV-2 RBD prior to transfusion (*n* = 25; blue). There were no statistically significant differences in any coronavirus antibodies before and after transfusion by a 2-way ANOVA (Tukey *post hoc*) of log-transformed values. *P* values of <0.05 were considered significant. Download FIG S1, DOCX file, 0.1 MB. Copyright © 2023 Karaba et al. 2023 Karaba et al. https://creativecommons.org/licenses/by/4.0/ This content is distributed under the terms of the Creative Commons Attribution 4.0 International license.

The increase in convalescent antibody levels against HKU1 and OC43 seen in this study, both of which are also betacoronaviruses, might be from cross-reactivity with elevated SARS-CoV-2 spike antibodies or induction of ehCoV-specific B cells to generate more ehCoV antibodies. We added purified SARS-CoV-2 spike at 150 μg spike/mL plasma (4) to donor unit plasma samples. SARS-CoV-2-specific antibodies were reduced 86% and HKU1 by 21%, suggesting minimal measurable cross-reactivity with HKU1 spike only (Fig. S2).

10.1128/mbio.03287-22.2 FIG S2 Competition of antibody signal with recombinant SARS-CoV-2 full-length spike protein: 13 donor units (red, no competition) were preincubated with 150 mcg recombinant WA-1 SARS-CoV-2 spike (4) per milliliter of plasma, followed by antibody level determination (black, competition). SARS-CoV-2 was significantly reduced by 86%, while HKU1 was nonsignificantly reduced by 21%, and 229E, NL63, and OC43 were unchanged. Download FIG S2, DOCX file, 0.1 MB. Copyright © 2023 Karaba et al. 2023 Karaba et al. https://creativecommons.org/licenses/by/4.0/ This content is distributed under the terms of the Creative Commons Attribution 4.0 International license.

Heterogeneity heat maps of antibody levels sorted by 229E indicated similar patterns among those hospitalized to those not hospitalized (Fig. S3). The donor CCP and control plasma had similar antibody levels despite regional donations (Fig. S4). Despite minimum ongoing ehCoV transmission due to social isolation measures during the trial, over time the remnant donor CCP and control plasma, as well as, in participants, the four ehCoV antibody levels, were the same over the more than 12 months of the study (Fig. S5 to S8). We observed no significant post-transfusion ehCoV antibody level changes in either the CCP or control plasma recipients to account for intervention arm differences in hospitalization from either ehCoV antibody COVID-19 cross-protection or enhancement. Many other studies measured 229E, OC43, NL63, and HKU1 antibody levels weeks after illness rather than during the first week of illness. The first week of illness had a higher predictive value on early hospitalizations, which mostly happened within a week of transfusion or within 2 weeks of illness onset.

10.1128/mbio.03287-22.3 FIG S3 Heterogeneity of viral antibody levels at the individual level with arbitrary units per milliliter depicted on log scale. (A) Antibody levels at enrollment (screening) for the 50 participants who were hospitalized, including those who received CCP or control plasma, sorted ascending by measured 229E. (B) Screening antibody levels for the 50 antibody-positive, vaccine-negative participants, including those who received CCP or control plasma, sorted ascending by measured 229E. (C) Screening antibody levels for the 100 antibody-negative, vaccine-negative participants who received control plasma, sorted ascending by measured 229E. (D) Screening antibody levels for the 100 antibody-negative, vaccine-negative participants who received CCP, sorted ascending by measured 229E. Download FIG S3, DOCX file, 0.2 MB. Copyright © 2023 Karaba et al. 2023 Karaba et al. https://creativecommons.org/licenses/by/4.0/ This content is distributed under the terms of the Creative Commons Attribution 4.0 International license.

10.1128/mbio.03287-22.4 FIG S4 CCP and control plasma donors segregated by region of donation by the 4 ehCoV. CCP are indicated by red circles and control plasma by blue circles. CCP: Washington DC and Maryland (*n* = 20), Iowa and Illinois (*n* = 13), and New York and New Jersey (*n* = 38). Control plasma: Washington DC (*n* = 45) and New Jersey (*n* = 5). Download FIG S4, DOCX file, 0.1 MB. Copyright © 2023 Karaba et al. 2023 Karaba et al. https://creativecommons.org/licenses/by/4.0/ This content is distributed under the terms of the Creative Commons Attribution 4.0 International license.

10.1128/mbio.03287-22.5 FIG S5 Individual CCP donors, sorted by date of donation during the collection period of the early treatment trial from 1 May to 30 September, for 229E (A), HKU1 (B), NL63 (C), and OC43 (D), with arbitrary units (AU) shown per milliliter. Download FIG S5, DOCX file, 0.09 MB. Copyright © 2023 Karaba et al. 2023 Karaba et al. https://creativecommons.org/licenses/by/4.0/ This content is distributed under the terms of the Creative Commons Attribution 4.0 International license.

The antibody levels of ehCoV in COVID-19 convalescent plasma were only 2-fold different between the control and convalescent plasma. The volume of distribution of the plasma was 3 to 6 L. The measured fold dilution when transfused into healthy individuals was 25-fold with a 200-mL CCP transfusion (15). When plasma with ehCoV antibodies was diluted 20-fold, it resulted in no change before or after transfusion, as shown here in over 300 paired samples, of which 51 donors were later hospitalized. Likewise, in those clinical trial recipients who were SARS-CoV-2 antibody negative or positive, there was no change in the levels before or after transfusion, but those who were seropositive for SARS CoV-2 at enrollment had HKU1 and OC43 levels that were twice as high compared to the seronegative participant ehCoV levels, which mirrored the levels seen in donor CCP compared to control plasma, with only the 2-fold difference, which, again, did not change the post-transfusion antibody levels.

Measurement of the available 17 hospitalized participants in the COVID-19 convalescent plasma arm and the 34 in the control plasma arm indicated no absolute ehCoV antibody level differences between these two groups or with 250 other paired participants. Even combining all 51 hospitalized participants to compare them to the 250 participants not hospitalized at screening before transfusion showed no differences.

In this study, the EhCoV antibody levels of those hospitalized and those not hospitalized were not distinguishable during the first week of illness, at which time there exists greatest risk for impact on severe COVID-19 outcome. Thus, it is unlikely that preexisting ehCoV antibodies in plasma have any clinically relevant impact on COVID-19 outcomes.

### Data availability.

The data sets generated during and/or analyzed during the current study are available from the corresponding author upon reasonable request.

10.1128/mbio.03287-22.6 FIG S6 Individual control plasma, sorted by date of donation during the collection period of the early treatment trial from 1 May to 30 September, for 229E (A), HKU1 (B), NL63 (C), and OC43 (D), with arbitrary units (AU) shown per milliliter. Download FIG S6, DOCX file, 0.07 MB. Copyright © 2023 Karaba et al. 2023 Karaba et al. https://creativecommons.org/licenses/by/4.0/ This content is distributed under the terms of the Creative Commons Attribution 4.0 International license.

10.1128/mbio.03287-22.7 FIG S7 Participants randomized to CCP with screening antibodies for ehCoV, sorted by date of enrollment during the collection period of the early treatment trial from 1 May to 30 September, for 229E (A), HKU1 (B), NL63 (C), and OC43 (D), with arbitrary units (AU) shown per milliliter. Download FIG S7, DOCX file, 0.1 MB. Copyright © 2023 Karaba et al. 2023 Karaba et al. https://creativecommons.org/licenses/by/4.0/ This content is distributed under the terms of the Creative Commons Attribution 4.0 International license.

10.1128/mbio.03287-22.8 FIG S8 Participants randomized to control plasma with screening antibodies against ehCoV, sorted by date of enrollment during the collection period of the early treatment trial from 1 May to 30 September, for 229E (A), HKU1 (B), NL63 (C), and OC43 (D), with arbitrary units (AU) shown per milliliter. Download FIG S8, DOCX file, 0.1 MB. Copyright © 2023 Karaba et al. 2023 Karaba et al. https://creativecommons.org/licenses/by/4.0/ This content is distributed under the terms of the Creative Commons Attribution 4.0 International license.
