# Erratum for Karaba et al., “Endemic Human Coronavirus Antibody Levels Are Unchanged after Convalescent or Control Plasma Transfusion for Early Outpatient COVID-19 Treatment”

**DOI:** 10.1128/mbio.00572-24

**Published:** 2024-03-21

**Authors:** Andrew H. Karaba, Trevor S. Johnston, Evan Beck, Oliver Laeyendecker, Andrea L. Cox, Sabra L. Klein, David J. Sullivan, COVID-19 Serologic Studies Consortium

## ERRATUM

Volume 14, no. 1, e03287-22, 2023, https://doi.org/10.1128/mbio.03287-22. Page 6, Acknowledgments paragraph 3, sentence 1, “K08AI156021, U54CA260491, and R01AI138897” should read “K08AI156021, and R01AI138897” and “(NCI) (U54CA260491)” should read “(NCI) (U54CA260492).”

