# Supplementary material for: Endemic Human Coronavirus Antibody Levels Are Unchanged after Convalescent or Control Plasma Transfusion for Early Outpatient COVID-19 Treatment
Source: mBio. 2023 Jan 10;14(1):e03287-22. doi: 10.1128/mbio.03287-22 (PMC9973272; doi:10.1128/mbio.03287-22)
Supplement: TABLE S1 [file mbio.03287-22-s0009.docx]

**Supplement Table 1** Substudy participant characteristics compared to parent study(1)

| Characteristic | Full Early treatment study  n (%) | Substudy on ehCoV  n (%) |
| --- | --- | --- |
| Number | 1181 | 250 |
| Median age (IQR) | 43 (32-54) | 48 (35-59) |
| Female | 675 (57) | 132 (53) |
| Age 18-34 | 355 (30) | 57 (23) |
| Age 35-49 | 415 (35) | 72 (29) |
| Age 50-64 | 331 (28) | 94 (38) |
| Age 65+ | 80 (7) | 26 (10) |
| BMI >=30 | 444 (37) | 92 (38) |
| BMI missing | 58 (5) | 10 (4) |
| Hypertension | 276 (23) | 65 (26) |
| Diabetes | 99 (8) | 20 (8) |
| Lung disease | 135 (11) | 27 (11) |
| HIV infection | 25 (2) | 10 (4) |
| Partial or full vaccination | 217 (18) | 0 (0) |

1. Sullivan DJ, Gebo KA, Shoham S, Bloch EM, Lau B, Shenoy AG, Mosnaim GS, Gniadek TJ, Fukuta Y, Patel B, Heath SL, Levine AC, Meisenberg BR, Spivak ES, Anjan S, Huaman MA, Blair JE, Currier JS, Paxton JH, Gerber JM, Petrini JR, Broderick PB, Rausch W, Cordisco ME, Hammel J, Greenblatt B, Cluzet VC, Cruser D, Oei K, Abinante M, Hammitt LL, Sutcliffe CG, Forthal DN, Zand MS, Cachay ER, Raval JS, Kassaye SG, Foster EC, Roth M, Marshall CE, Yarava A, Lane K, McBee NA, Gawad AL, Karlen N, Singh A, Ford DE, Jabs DA, Appel LJ, Shade DM, et al. 2022. Early Outpatient Treatment for Covid-19 with Convalescent Plasma. N Engl J Med doi:10.1056/NEJMoa2119657.
