# Supplementary material for: Endemic Human Coronavirus Antibody Levels Are Unchanged after Convalescent or Control Plasma Transfusion for Early Outpatient COVID-19 Treatment
Source: mBio. 2023 Jan 10;14(1):e03287-22. doi: 10.1128/mbio.03287-22 (PMC9973272; doi:10.1128/mbio.03287-22)
Supplement: FIG S1 [file mbio.03287-22-s0001.docx]

**Supplement Figure 1** **A.** Coronavirus antibody levels (AU/mL) before and 30 minutes after transfusion of CCP in participants who were seropositive for SARS-CoV-2 RBD prior to transfusion (n = 25, red). **B.** Coronavirus antibody levels (AU/mL) before and 30 minutes after transfusion of control plasma in participants who were seropositive for SARS-CoV-2 RBD prior to transfusion (n = 25, blue). There were no statistically significant differences in any coronavirus antibodies before and after transfusion by a 2-way-Anova (Tukey post hoc) of log transformed values. P-values < 0.05 were considered significant.
