# Supplementary material for: Endemic Human Coronavirus Antibody Levels Are Unchanged after Convalescent or Control Plasma Transfusion for Early Outpatient COVID-19 Treatment
Source: mBio. 2023 Jan 10;14(1):e03287-22. doi: 10.1128/mbio.03287-22 (PMC9973272; doi:10.1128/mbio.03287-22)
Supplement: FIG S2 [file mbio.03287-22-s0002.docx]

**Supplement Figure 2** Competition of antibody signal with recombinant SARS-CoV-2 full length spike protein. 13 donor units (red, no competition) were preincubated with 150 mcg recombinant WA-1 SARS-CoV-2 spike(1)/mL of plasma followed by antibody level determination (black, competition). SARS-CoV-2 was significantly reduced 86%, while HKU1 was non-significantly reduced 21% and 229E, NL63 and OC43 were unchanged.

1. Klein S, Pekosz A, Park H-S, Ursin R, Shapiro J, Benner S, Littlefield K, Kumar S, Naik HM, Betenbaugh M, Shrestha R, Wu A, Hughes R, Burgess I, Caturegli P, Laeyendecker O, Quinn T, Sullivan D, Shoham S, Redd A, Bloch E, Casadevall A, Tobian A. 2020. Sex, age, and hospitalization drive antibody responses in a COVID-19 convalescent plasma donor population. J Clin Invest 130:6141-6150.
