# Supplementary material for: Endemic Human Coronavirus Antibody Levels Are Unchanged after Convalescent or Control Plasma Transfusion for Early Outpatient COVID-19 Treatment
Source: mBio. 2023 Jan 10;14(1):e03287-22. doi: 10.1128/mbio.03287-22 (PMC9973272; doi:10.1128/mbio.03287-22)
Supplement: FIG S3 [file mbio.03287-22-s0003.docx]

**Supplement Figure 3** Heterogeneity of viral antibody levels at the individual level with arbitrary units per mL depicted on log scale**. A)** Antibody levels at enrollment (screening) for the 50 participants who were hospitalizations including those who received CCP or control plasma sorted ascending by measured 229E. **B)** Screening antibody levels for the 50 antibody positive, vaccine negative participants including those who received CCP or control plasma sorted ascending by measured 229E. **C)** Screening antibody levels for the 100 antibody negative , vaccine negative participants in those who received control plasma sorted ascending by measured 229E**. D)** Screening antibody levels for the 100 antibody negative , vaccine negative participants in those who received CCP sorted ascending by measured 229E.
