# Supplementary material for: Endemic Human Coronavirus Antibody Levels Are Unchanged after Convalescent or Control Plasma Transfusion for Early Outpatient COVID-19 Treatment
Source: mBio. 2023 Jan 10;14(1):e03287-22. doi: 10.1128/mbio.03287-22 (PMC9973272; doi:10.1128/mbio.03287-22)
Supplement: FIG S4 [file mbio.03287-22-s0004.docx]

**Supplement Figure 4.** CCP and Control plasma donors segregated by region of donation by the 4 ehCoV. CCP are red circles and control plasma are blue circles. CCP Washington DC and Maryland (n=20), Iowa and Illinois (n=13), New York and New Jersey (n=38). Control plasma Washington DC (n=45) and New Jersey (n=5).
