# Supplementary material for: Endemic Human Coronavirus Antibody Levels Are Unchanged after Convalescent or Control Plasma Transfusion for Early Outpatient COVID-19 Treatment
Source: mBio. 2023 Jan 10;14(1):e03287-22. doi: 10.1128/mbio.03287-22 (PMC9973272; doi:10.1128/mbio.03287-22)
Supplement: FIG S6 [file mbio.03287-22-s0006.docx]

**Supplement Figure 6** Individual control plasma sorted by date of donation during the collection period of the early treatment trial from May 1 to September 30. **A)** 229E **B)** HKU1 **C)** NL63) and **D)** OC43 with arbitrary units shown per mL (AU per mL).
